# Optimal neuromonitoring techniques in neonates with hypoxic ischemic encephalopathy

**DOI:** 10.3389/fped.2023.1138062

**Published:** 2023-03-08

**Authors:** Valerie Y. Chock, Anoop Rao, Krisa P. Van Meurs

**Affiliations:** Division of Neonatal and Developmental Medicine, Department of Pediatrics, Stanford University School of Medicine and Lucile Packard Children's Hospital Stanford, Palo Alto, CA, United States

**Keywords:** neuromonitoring, near-infrared spectroscopy, amplitude integrated electroencephalography, heart rate variability, visual evoked potentials, somatosensory evoked potentials, hypoxic ischemic encephalopathy

## Abstract

Neonates with hypoxic ischemic encephalopathy (HIE) are at significant risk for adverse outcomes including death and neurodevelopmental impairment. Neuromonitoring provides critical diagnostic and prognostic information for these infants. Modalities providing continuous monitoring include continuous electroencephalography (cEEG), amplitude-integrated electroencephalography (aEEG), near-infrared spectroscopy (NIRS), and heart rate variability. Serial bedside neuromonitoring techniques include cranial ultrasound and somatic and visual evoked potentials but may be limited by discrete time points of assessment. EEG, aEEG, and NIRS provide distinct and complementary information about cerebral function and oxygen utilization. Integrated use of these neuromonitoring modalities in addition to other potential techniques such as heart rate variability may best predict imaging outcomes and longer-term neurodevelopment. This review examines available bedside neuromonitoring techniques for the neonate with HIE in the context of therapeutic hypothermia.

## Introduction

Neonatal hypoxic ischemic encephalopathy (HIE) continues to be a significant health problem leading to death and long-term disability. Randomized controlled trials have evaluated the effect of systemic hypothermia on newborns with moderate to severe HIE and the results are encouraging; however, the incidence of death or moderate to severe disability remains 44%–52% in infants receiving induced hypothermia (1). A major practical and ethical dilemma is the challenge of determining in real time the extent of neuronal loss and irreversible injury which may have occurred to help guide discussions with families. Methods available for neurological assessment include clinical data, neurologic examination, neuroimaging, and neurodiagnostic techniques, but determination of which method is both highly predictive of death and disability, and feasible during the first few hours and days of life has been challenging. A reliable and accurate bedside technique is needed to assist in predicting outcome early in the neonatal course to better inform decision making by physicians and parents.

The randomized controlled trials of therapeutic hypothermia for newborns with HIE marked the beginning of active investigation into the use of continuous neuromonitoring techniques in the neonatal intensive care unit (NICU). Prior to this period, video EEG was used in the NICU, but rarely for prolonged periods of time. The primary focus has been identification of electrographic seizure activity. For prediction of outcome, EEG background pattern is the most important factor and EEGs with low voltage, persistent burst suppression, or electrocerebral inactivity are highly correlated with adverse outcome and death. Amplitude integrated electroencephalography (aEEG), a simplified, continuous EEG using a small number of electrodes provides an overall impression of cerebral activity. The cerebral function monitor (CFM) was developed by Prior and Maynard in the 1960s for use in adults and subsequently applied to newborns in the late 1970s and 1980s. Its use has become more widespread for outcome prediction as well as for seizure detection particularly in newborns with perinatal asphyxia. The terminology used in aEEG is similar to EEG allowing for communication between neonatologists and neurologists.

Near infrared spectroscopy (NIRS) is a more recently utilized technology for continuous, bedside, neuromonitoring by measuring trends in cerebral oxygenation and evaluating the balance between oxygen delivery and consumption. NIRS monitors also demonstrate the relationship between oxygenation of the brain and other end organs by providing cerebral and somatic oximetry values. Heart rate variability has also emerged as a potential bedside measure which may be predictive of outcomes in the HIE population. Other bedside neuromonitoring devices used less frequently in the NICU include visual and somatosensory evoked potentials.

This manuscript will discuss the evidence for the use of these neuromonitoring techniques in newborns with HIE focusing primarily on outcome prediction and seizure detection.

## Continuous electroencephalography (cEEG)

Electroencephalography is the recording of brain waves allowing for measurement of brain function. In newborns, electrode placement is according to the International 10–20 System, modified for the smaller head size. In a term infant, 9–11 electrodes are used whereas in a preterm newborn fewer are used. Electrode placement is at specific positions on the scalp and for this reason an EEG technologist is necessary. Interpretation is performed by a neurophysiologist trained in EEG interpretation. When the EEG is for a short period such as 30–60 min it is considered a routine or “spot” EEG while longer recordings are termed continuous EEG (cEEG) and record for 24 h or more, frequently including video (vEEG). cEEG is a key neuromonitoring tool when caring for critically ill term and preterm neonates. Its primary roles are identification of electrographic seizure activity and prognostication by assessing the background brain activity. The American Clinical Neurophysiology Society has written guidelines for cEEG monitoring in neonates (2). These guidelines describe preferred methods and indications; however, these guidelines are not a standard that all centers can achieve due to the personnel and equipment requirements. The guidelines list the diagnoses with a high risk for seizures where long-term EEG monitoring should be considered; neonatal depression due to suspected perinatal asphyxia is listed first. The guidelines recommend monitoring with cEEG for 24 h to screen for seizures and if seizures are detected continuing for at least 24 h following the last electrographic seizure. If suspicious clinical events are occurring, monitoring should continue until multiple typical events are captured without an associated electrographic seizure.

### Identification of electrographic seizure activity

EEG is accepted as the gold standard for seizure detection and cEEG is felt to be superior to the use of spot EEG. Both the high rate of subclinical seizures in neonates without any clinical correlate, as well as the fact that unusual movements such as bicycling or lip smacking are not always accompanied by electrographic findings make cEEG essential in the management of seizures in neonates (3). If only clinical seizures are treated, this can lead to under-diagnosis of subclinical seizures as well as the over-treatment of suspicious clinical events. Once anti-epileptic drugs have been used it is well recognized that uncoupling of electrographic and clinical findings is common, further increasing the value of cEEG in management of neonatal seizures (4). Two randomized controlled trials have suggested that outcomes are improved by treating subclinical seizures in newborns with HIE (5, 6). Furthermore, several studies have demonstrated that the use of cEEG within a neonatal neurocritical care setting with a seizure guideline has reduced the dosage and duration of anti-epileptic drugs used, either confirmed or ruled out seizures in more than one-third of cases and reduced the number of neonates progressing to status epilepticus (7–10). These improvements are seen as important as the overall seizure burden is associated with death and disability. Furthermore, seizures adversely impact the developing brain. Glass et al. found that clinical seizure activity in neonates with HIE is associated with worse neurodevelopmental outcome after controlling for the severity of brain injury on MRI (11). This suggests that optimal management of seizures in neonates with HIE may improve neurodevelopmental outcomes. During therapeutic hypothermia, electrographic seizure activity is seen in 31 to 64% of neonates with HIE and the majority of seizures occur within 24 h after birth (12, 13). A recent investigation by Chalak et al. found that there was a higher odds of seizures during the re-warming period when compared to a preceding epoch (14). Seizures are also more likely in newborns with a background pattern showing flat tracing. For this reason, the current recommendation is to initiate cEEG shortly after birth and to continue through the entire period of cooling and re-warming.

### Prognostication of outcome

EEG is frequently used when assessment of brain function is needed; often when a neonate is encephalopathic or the neurologic exam is otherwise worrisome. EEG background activity has been found to prognosticate outcome in newborns with HIE and serial EEG can be used to evaluate changes over time. Normal background in a term infant is a continuous, symmetric mixture of normal amplitude activity. During sleep, there are alternating higher and lower amplitudes termed trace alternant. Excessive discontinuity in a term infant is abnormal and reflects abnormal brain function due to underlying pathology. Discontinuity is often seen in HIE and the degree of discontinuity reflects the severity of injury (15). Mild or moderate discontinuity may be seen with the use of anticonvulsant or sedative medications. Extremely low amplitude activity or burst suppression patterns are commonly associated with very poor prognosis. Asymmetry of the EEG pattern can indicate lateralized brain injury and warrants neuroimaging to look for a structural cause such as hemorrhage or stroke. As discussed previously, the presence of clinical seizures has been independently associated with worse neurodevelopmental outcome (11). In addition, in a multi-center observational study of newborns with seizures where 38% of cases were due to HIE, high electrographic seizure burden was a significant risk factor for mortality, length of hospital stay, and abnormal neurologic exam at discharge (16). The prognostic value of cEEG to predict Bayley-III cognitive, motor, and language outcomes at 24 months of age was evaluated in 41 neonates treated with therapeutic hypothermia for HIE (17). The authors found that higher power of central and occipital cortical bursts predicted worse cognitive and language outcome while higher power of central cortical bursts predicted worse motor outcome. A lower seizure burden has been reported in newborns with moderate HIE treated with therapeutic hypothermia and this finding may partially explain the therapeutic benefit (18).

## Amplitude integrated electroencephalography (aEEG)

aEEG is a bedside brain monitoring tool which is used with increasing frequency in NICUs worldwide. It was first developed as a tool to assess the depth of anesthesia during surgery. The aEEG data is derived following a process where the raw EEG tracing is filtered, amplified, rectified, and then displayed in a time-compressed, semi-logarithmic fashion. Current aEEG devices display the compressed as well as the raw EEG tracing allowing for evaluation of the background activity of the brain, displaying changes in the background activity over time, and enabling screening for seizure activity.

**Figure 1:**
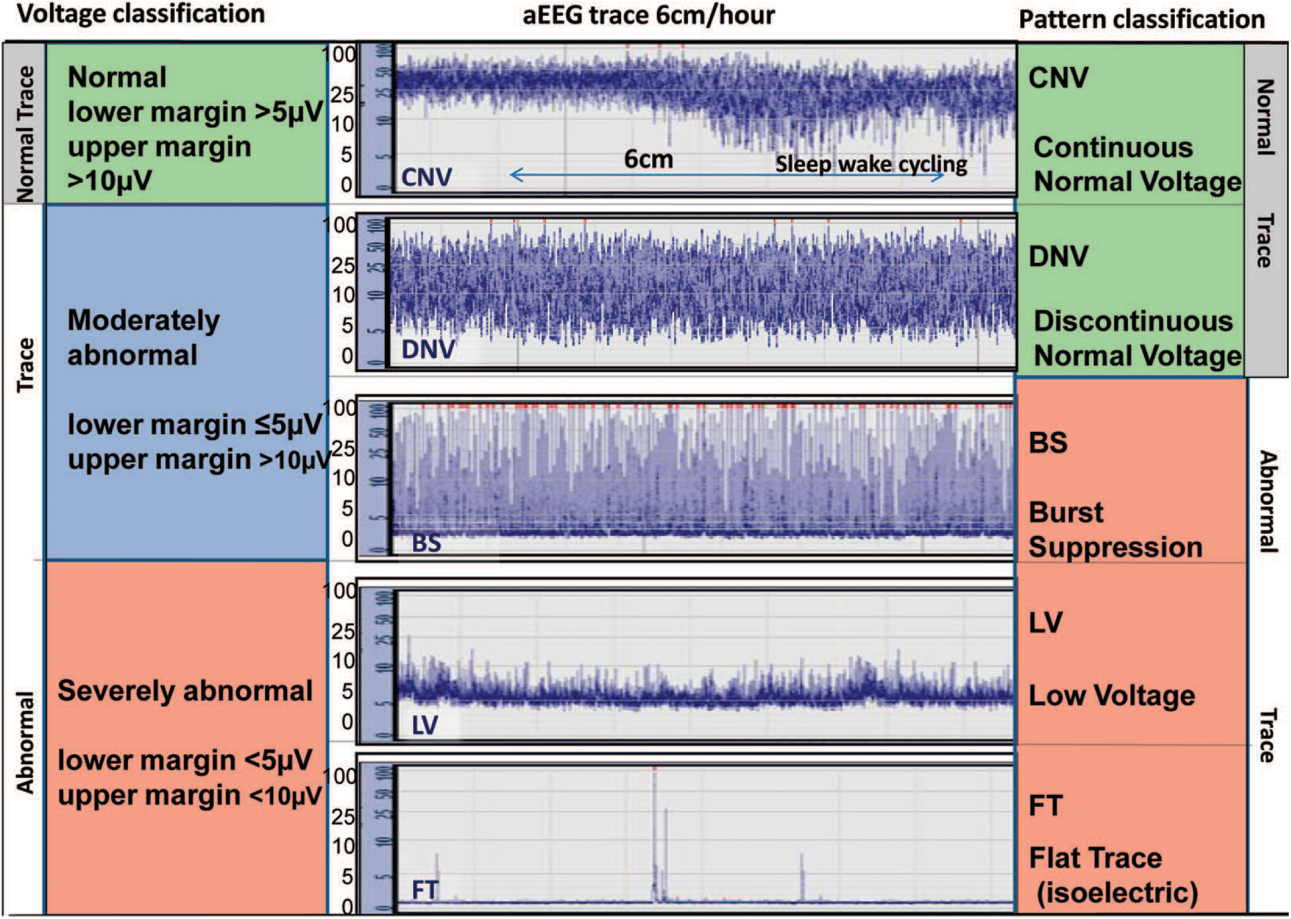
Classification systems for aEEG background pattern: Voltage method on left and pattern recognition on right (reproduced with permission from Hellstrom-Westas L. Continuous electroencephalography monitoring of the preterm infant. Clin Perinatol. 2006;33(3):633–47, vi).

**Figure 2:**
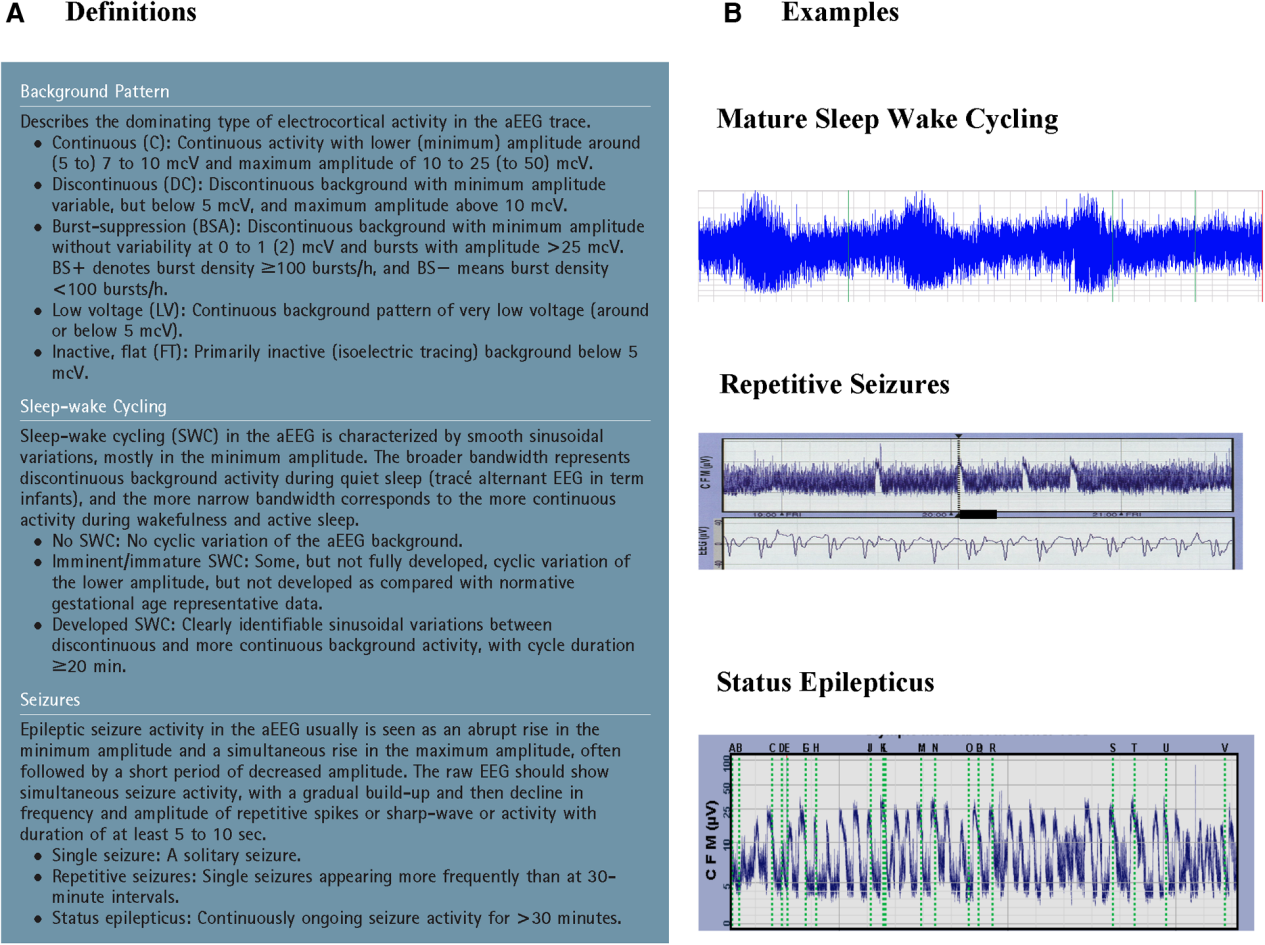
A: Suggested classification of aEEG patterns in preterm and term infants (reproduced with permission from hellström-westas L, rosén I, de Vries L, greisen G. Amplitude-integrated EEG classification and interpretation in preterm and term infants. Neoreviews. 2006;7(2):376-e86) and B: Examples of sleep-wake cycling, repetitive seizures, and status epilepticus.

The first aEEG background classification system was developed by Hellström-Westas and colleagues (19) (Figure 1). It is based on pattern recognition and uses conventional EEG terminology which facilitates communication between neonatologists and neurologists. A second classification system developed by al Naqeeb uses voltage-based criteria and is also shown in Figure 1 (20). Figure 2 describes the 5 background patterns and their voltage criteria as well as the classifications often used for sleep wake cycling (SWC), and seizures (21). Medications such as sedatives, analgesics, and anticonvulsants may have an impact on the background pattern with lower amplitudes or alterations in SWC. Furthermore, there are also potential sources of artifact which may affect an aEEG recording. Some are electrical from sources such as high frequency ventilation, electrocardiogram (ECG), electromyography (EMG), or other medical equipment, while others are from physical movement of the neonate such as hiccups, patting, and sucking.

aEEG has a number of strengths as the leads are easy to apply and do not require an EEG technician. Furthermore, aEEG devices are easy to use and do not interfere with bedside care. Interpretation does not require extensive training in neurophysiology. Both physicians and nurses can be trained to initiate aEEG monitoring and recognize and document the background pattern and areas of the tracing concerning for seizure; However, aEEG does not replace cEEG as the “gold standard” for seizure detection. Seizure diagnosis is facilitated by evaluation of both the compressed aEEG as well as the raw EEG trace and review by an experienced provider is critical. As aEEG only records from a limited number of channels, seizures arising away from the central and parietal regions where the electrodes are usually located can be missed. Seizures which are brief (less than 30 s), focal, or low amplitude are not recognized as they are not visible on the compressed tracing.

### Prognostication of outcome

**Figure 3:**
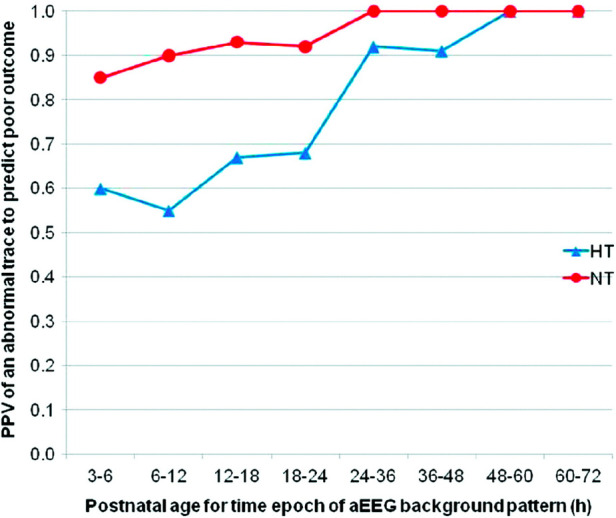
The PPV for an abnormal tracing to predict adverse outcome. HT = hypothermia and NT = normothermia. Abnormal tracing Is defined as burst suppression, low voltage or flat tracing and adverse outcome Is defined as death or Either Bayley Scales of Infant Development Mental Developmental Index <70, gross motor classification level 3-5 or No useful vision. (reproduced with permission from thoresen M, hellström-westas L, Liu X, de Vries LS. Effect of hypothermia on amplitude-integrated electroencephalogram in infants with asphyxia. Pediatrics. 2010 Jul;126(1):e131-139).

Evolution of the aEEG background pattern in term neonates with HIE has been known to convey prognostic information dating back to a study by Bjerre et al. in 1983 (22). A subsequent study by ter Horst et al. focused on 30 term newborns with severe asphyxia and examined the aEEG tracing over the first 72 h of life using pattern recognition (23). The evolution of the aEEG pattern in relation to the neurologic outcome was examined and the sooner the aEEG changed to a normal pattern, either continuous normal voltage or discontinuous normal voltage, the better the prognosis. Normal patterns by 48 h of age were associated with normal outcome while burst suppression or worse was associated with adverse outcome. Early aEEG tracings were used in 3 of the randomized trials as an entry criterion due to its prognostic accuracy within several hours after birth (80%–85% within 6 h after birth) (24–26). To determine the effect of hypothermia treatment on the predictive value of aEEG in newborns with HIE, Thoresen et al. compared the predictive value of aEEG performed at <6 h on outcome as measured by Bayley Scales of Infant Development II at 18 months of age in normothermia (*n* = 31) and hypothermia treated infants (*n* = 43) (27). Tracings with continuous normal voltage (CNV) or discontinuous normal voltage (DNV) were classified as normal while burst suppression (BS), continuous low voltage (CLV) or flat tracing (FT) were classified as abnormal. The authors found that the positive predictive value of an abnormal aEEG pattern at 3–6 h of age was 84% with normothermia compared to 59% for hypothermia. Time to normal background pattern was the best predictor of poor outcome (96.2% with hypothermia) and never developing SWC always predicted poor outcome. They concluded that early aEEG patterns do not accurately predict outcome in neonates treated with hypothermia and that infants with good outcome had normal background patterns return by 48 h of age (Figure 3). Several single center studies have confirmed these findings. Sewell et al. looked at background pattern evolution and the specific hour of life where the aEEG normalized or sleep wake cycling developed (28). They categorized evolution into 6 separate patterns and concluded that this classification distinguished between outcome groups more reliably, evidenced by a higher likelihood ratio compared with assessment of the background pattern at discrete timepoints. Newborns who did not have a discontinuous pattern by 15.5 h of life, cycling by 45.5 h or continuous normal voltage by 78 h of life were most likely to have an adverse outcome, defined as death or severe brain injury on MRI.

**Figure 4:**
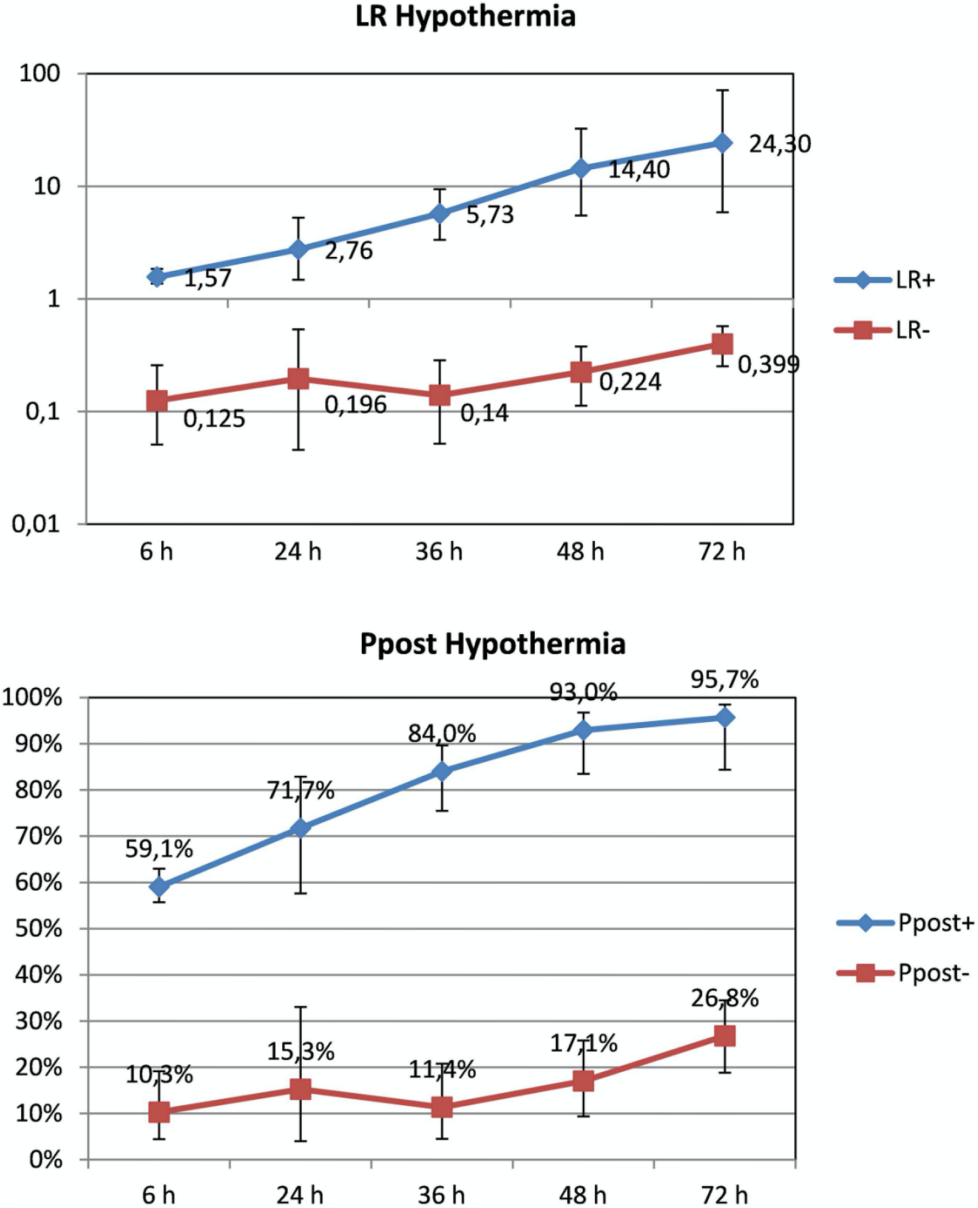
Likelihood ratios (LR) and post-test probability (ppost) of abnormal aEEG and adverse neurologic outcome in newborns treated with hypothermia at specific hours of life (reproduced with permission from Del Río R, ochoa C, alarcon A, arnáez J, blanco D, garcía-alix A. Amplitude Integrated Electroencephalogram as a Prognostic Tool in Neonates with Hypoxic-Ischemic Encephalopathy: A Systematic Review. PLoS ONE. 2016;11(11):e0165744).

Several meta-analyses have sought to further clarify the role of aEEG in prediction of outcome for HIE. Each have had a slightly different focus. A study by Spitzmiller in 2007 was the first meta-analysis to evaluate the use of aEEG to predict outcome in HIE prior to the use of therapeutic hypothermia (29). In a pooled analysis of 8 studies including 529 newborns, the sensitivity of an abnormal aEEG tracing for prediction of poor outcome was 91% (95% Confidence Interval (CI) 87%–95%) with specificity 88% (95% CI 84%–94%). They noted that infants who progressed to a normal tracing earlier (8–12 h of age) had better outcome than those infants whose tracings remained abnormal or worsened during the monitoring period. A meta-analysis by Awal et al. examined which background patterns most accurately predicted long-term outcome (30). They included 31 studies and concluded that burst suppression, low voltage, and flat tracing had the highest odds ratios for abnormal outcome (Table 1). Limitations were the variability in the background pattern definitions and the type of aEEG device used. In 2016, del Rio et al. published a systematic review to determine the optimal chronologic age to predict outcome in neonates with HIE treated with TH. Seventeen studies and 360 infants were analyzed. They concluded that the maximum predictive reliability was achieved at 72 h (post-test probability 95.7%, 95% CI 84%–98%) while the predictive value at 6 h was low (59%, 95% CI 55%–63%) (Figure 4) (31). Chandrasekaran et al. also investigated the optimal timing for prognostication and compared timepoints at 6, 24, 48 and 72 h of age including 9 studies and 520 infants in this analysis (32). The odds ratio was highest at 48 h of age (66.9 with 95% CI 19.7–227.2). Prior to the use of therapeutic hypothermia, an abnormal aEEG at 6 h of age was predictive of abnormal outcome (33). Due to the beneficial effects of therapeutic hypothermia performed over a 72 h period, the maximum predictive value has shifted and is now seen at 48–72 h of age.

**Table 1:** Sensitivity and specificity for abnormal background patterns to predict adverse outcome.

aEEG Background pattern	No. studies	No. subjects	Sensitivity Point estimate (95% CI)	Specificity Point estimate (95% CI)
Burst suppression	29	914	0.87 (0.78–0.92)	0.82 (0.72–0.88)
Low voltage	19	566	0.92 (0.72–0.98)	0.99 (0.87–1.0)
Flat/inactive	13	493	0.78 (0.58–0.91)	0.99 (0.88–1.0)

## Near infrared spectroscopy (NIRS)

As neonatal HIE may also result in hemodynamic and cerebral metabolic alterations, measurement of cerebral oxygenation (rScO2) and cerebral fractional tissue oxygen extraction (fTOE) with NIRS bedside monitoring has significant promise as a neurodiagnostic technique. A NIRS sensor placed on the left or right forehead emits near-infrared light, which penetrates through skin and bone and is differentially absorbed by oxygenated and deoxygenated hemoglobin in the underlying tissue. Residual light is then reflected back to a detector, with subsequent calculation of rScO2, a measure of tissue oxygenation. Cerebral fTOE is estimated using the combined measure of peripheral oxygen saturation (SpO2) and rScO2 (fTOE = (SpO2 – rScO2)/SpO2), indicating the balance between oxygen delivery and oxygen consumption in the interrogated tissue. Different commercial NIRS devices operate using these same principles, although with slightly different light wavelengths and algorithms, resulting in discrepancies in absolute rScO2 levels between devices (34). For this reason, trends in rScO2 may be of greater comparative significance. In the transition period after birth, rScO2 peaks by 36 h of age, but then declines over the first week as cerebral fTOE increases (35, 36). Nonetheless, population-based normative values range between 55%–85% in the preterm infant using a small adult sensor and INVOS device (Medtronic, Minneapolis, MN), with higher rScO2 seen with increasing gestational age (35–37). Term infants using the same NIRS device with pediatric sensors similarly had normative values 71%–85% in the first 2 days of age (38, 39).

**Figure 5:**
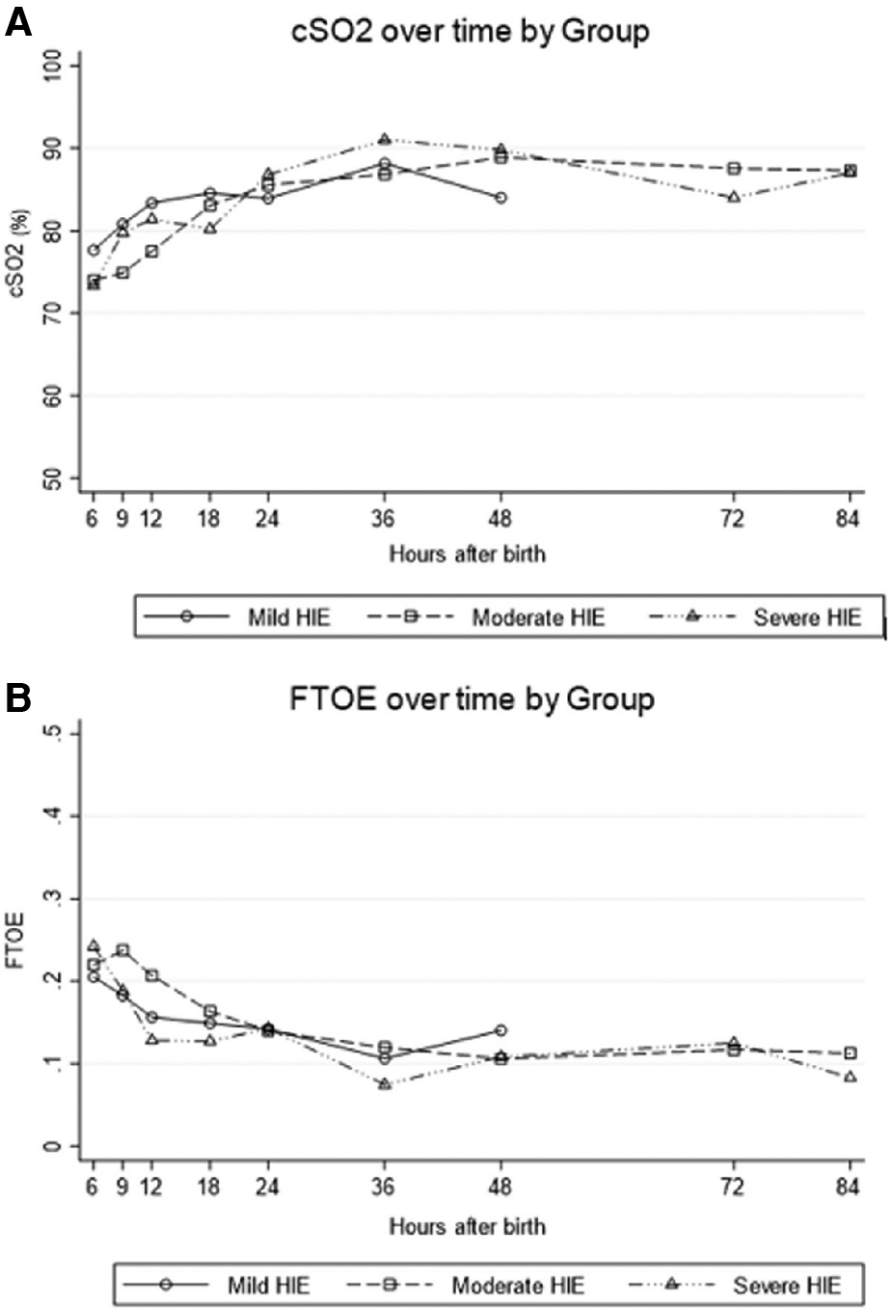
For All grades of neonatal encephalopathy, (**A**) cerebral saturation (cSO2) increases over the first 24 h of life and (**B**) cerebral fractional tissue oxygen extraction (FTOE) levels decrease (reproduced with permission from garvey AA, O’Toole JM, livingstone V, et al. Evolution of early cerebral NIRS in hypoxic ischemic encephalopathy. Acta Paediatr. 2022;111:1870-77).

**Figure 6:**
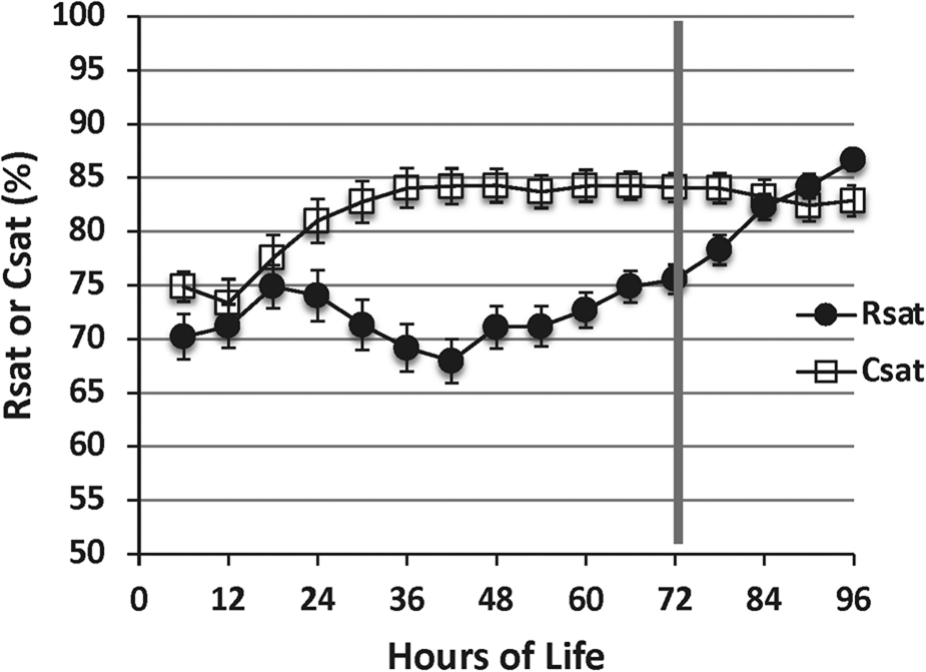
Cerebral saturation (csat) Is higher during the cooling period compared with renal saturation (rsat) (*p* < .01). After rewarming at 72 h, cerebral saturation decreases While renal saturation increases (reproduced with permission from Chock VY, Frymoyer A, Yeh CG, Van Meurs KP. Renal Saturation and Acute Kidney Injury in Neonates with Hypoxic Ischemic Encephalopathy Undergoing Therapeutic Hypothermia. J Pediatr. 2018;200:232-239.e1).

In infants with HIE, decreased cerebral blood flow after acute injury is followed by reperfusion over time. As a surrogate for cerebral blood flow, the chronologic evolution of rScO2 may be an important bedside measure and further serve as a prognostic tool (40). While therapeutic hypothermia itself contributes to vasoconstriction and decreased cerebral blood flow, this effect is mitigated by a larger decrease in metabolic demand, subsequently resulting in higher rScO2 and lower fTOE (41, 42) (Figure 5). After initiation of cooling, a modest increase in rScO2 is typically seen due to these effects. However, minimal changes after rewarming demonstrate that the impact of therapeutic hypothermia on the brain is not as significant compared to the effect of hypothermia on other organs such as the kidney (43). In a study by Chock, et al. mean rScO2 decreased non-significantly by 0.5% after rewarming compared to an increase in renal tissue saturation by 12 ± 8% after rewarming (Figure 6) (43).

### Prognostication of outcome

Several studies have examined cerebral NIRS measures during therapeutic hypothermia as a predictor for both adverse brain MRI outcomes and longer-term neurodevelopmental outcomes. High rScO2 and low fTOE are associated with worse neuroimaging outcomes by MRI in several small studies (44–47). A high rScO2 may indicate that cerebral blood flow occurs in excess of the need for oxygen utilization (luxury perfusion). Decreased cerebral oxygen utilization may also be a marker of secondary energy failure after neuronal cell death. Jain et al. found that in a small study of 21 neonates cooled for HIE, higher rSO2 between 24 and 36 h correlated with subcortical brain injury on MRI. Peng et al. found that for maximum rScO2 > 75% by as early as 10 h of age, the sensitivity for subsequent brain MRI injury was 100% and specificity 83% with AUC 0.93 (45). Szakmar, et al. showed association between rScO2 during rewarming and grey matter injury on post-rewarming MRI with adjusted odds ratio 1.23 (95% confidence interval 1.02–1.49) (47). In contrast, Shellhaas et al. did not find any correlation between cerebral NIRS measures at rewarming and MRI outcomes (48). The most predictive time period of NIRS monitoring varied between studies and may be related to MRI grading system, type of NIRS device, and time period of monitoring, although most support predictive capabilities by at least 24 h of age (44, 45).

Similarly, several small studies have demonstrated adverse neurodevelopmental outcomes at 18–30 months of age in infants with higher rScO2 and lower fTOE during cooling. These studies all were limited by relatively small sample size and differed on optimal time period to capture differences, with best predictive capabilities ranging from 12 h of age, 24–48 h of age, or after 72 h and rewarming (44, 49–51).

Rate of change of rScO2 may also be prognostic and associated with the severity of HIE. In a prospective, observational study, 15 non-cooled infants with mild HIE had significantly higher mean rScO2 and lower FTOE at 6 h of age compared to 15 cooled infants with moderate HIE (mean difference in rScO2 of 8.1% (95% confidence interval 2.7%–13.5%)), although differences resolved by 18 h (40). An early time-point of NIRS assessment such as 6 h of age may thus have very different implications than measurements at 24–48 h, with need for close evaluation of the trajectory of NIRS measures. Wintermark, et al. found a greater increase in rScO2 from day 1 to day 2 in four infants with severe HIE (mean increase of 4.4 ± 1.9%) compared to three with moderate HIE (mean increase of 1.3 ± 0.1%) (52). Jain et al., also found that those with more rapidly increasing rScO2 until 36 h of age had increased injury on brain MRI (44).

More sophisticated NIRS techniques and analyses of NIRS data may provide additional insight for the neonatal HIE population. Several investigators have explored impaired cerebral autoregulation with failure to regulate cerebral blood flow as a mechanism for brain injury after HIE. Various measures of autoregulation using NIRS have included a higher pressure passivity index with increased coherence between mean arterial pressure and cerebral oxygen saturation, increased time domain reactivity index, and wavelet coherence analysis (53–55). Regionality of impaired autoregulation has also been described, with greatest differences in anterior compared to posterior measures (56). These findings suggest that temporal-occipital regions may potentially be more vulnerable to autoregulatory impairment given higher baseline perfusion and metabolism in these areas in the newborn. The wavelet neurovascular bundle concept moreover combines metrics from NIRS, blood pressure, and EEG in neonates with HIE to measure changes in brain vascular function as related to both cerebral autoregulation and neuronal electrical activity (55). Others have combined frequency domain NIRS with diffusion correlation spectroscopy to calculate cerebral metabolic rate of oxygen (57). Ideally, future advances in NIRS monitoring may permit targeted strategies to improve outcomes in neonates with HIE.

## Heart rate variability

Perinatal oxygen deprivation with subsequent HIE may impact cortical and subcortical neuronal pathways, disrupting the integrity of the autonomic nervous system (58, 59). Consequently, autonomic influence over the sino-atrial node is deranged leading to alterations in heart rate variability (HRV). While core body temperature is a relevant factor and therapeutic hypothermia itself reduces heart rate and respiratory rate, persistent changes in HRV are reflective of the underlying pathophysiology (60, 61). Accordingly, HRV has been proposed as a potential biomarker for HIE severity during the first week of life (62). Of note, HRV is not a single entity but rather a set of linear (across time and frequency domains) and non-linear metrics. In this section, HRV predominantly refers to the time and frequency domain indices.

A synthesis of four observational studies (*n* = 248) concluded that moderate and severe HIE was associated with a reduction in most HRV measures (62). Generally, infants with HIE had significantly lower low frequency (LF), higher high frequency (HF) and lower LF/HF ratio and values compared with controls (63). This reflects an increased parasympathetic and decreased sympathetic drive (64). HRV parameters also correlated with severity of findings on multichannel EEG recordings in patients with HIE (65). From a prognostic perspective, a depressed HRV on DOL1 was associated with moderate-severe HIE and adverse outcomes including mortality (66). It has also been shown that the degree of HRV depression is related to the pattern of brain injury and topography of brain injury (67). Furthermore, HRV at 24 h of life and post-rewarming predicted severity of brain injury on MRI, death or impaired neurodevelopment at 15 months, and two years of life (65–68). Autonomic dysfunction was shown to impairment at school age in survivors of neonatal HIE (69). Of note, unlike cEEG, aEEG and NIRS, nearly all the work that relates to HRV in patients with HIE has been retrospective. Only one study used real-time assessment with a heart rate characteristic (HRC) index score and found that loss of HRV during therapeutic hypothermia was associated with severity of MRI injury after rewarming (70, 71). With regards to expected confounders, males generally had a higher HRV compared to females and mechanical ventilation did not significantly affect HRV, although the LF/HF ratio may be altered given the loss of respiratory variability in mechanically ventilated infants (58, 72). The impact of prematurity, concurrent sepsis, seizure activity, vasoactive medications and sedatives on HRV metrics in patients with HIE remains an active area of research. Nevertheless, despite inconsistencies of definitions and methodologies, prior research supports use of HRV metrics as a non-invasive adjunct in assessing patients with HIE. The ability to translate these findings for real-time monitoring to inform prognosis, evaluate strategies for neuroprotective intervention, and track recovery will be vital for impacting care of patients with HIE.

## Serial bedside neuromonitoring methods

### Cranial ultrasound

Serial cranial ultrasound studies may also be useful in evaluation of brain injury after neonatal HIE. For example, assessment of cerebral edema or increased echogenicity in vulnerable areas like basal ganglia may be determined. However, predictive accuracy is relatively low for abnormal neurologic outcome at 18 months (73). Moreover, disagreement exists over the value of early cranial ultrasound compared to more definitive MRI imaging after rewarming (74). Ongoing research into power Doppler ultrasound to calculate cerebral blood flow may improve use of this technique as a predictive tool, although its utility may be limited by discrete measurement intervals compared to continuous monitoring modalities (75).

### Visual and somatosensory evoked potentials

Evoked potentials are electrical responses to visual or somatosensory stimulation and may help in prognostication of outcomes in infants with HIE. Visual evoked potentials (VEP) are typically obtained to assess the optic pathway in response to a flash of light. Most studies demonstrating association of abnormal VEPs with adverse neurodevelopmental outcome were obtained in the pre-hypothermia era, although in more recent cohorts of infants treated with therapeutic hypothermia for HIE, VEP abnormalities have been associated with impaired hearing-language scores and with abnormal glucose levels and MRI brain injury (76–78). Somatosensory evoked potentials (SEP) are measured by electric stimulation of both median nerves to assess function of the sensory cortex and sensory pathways. Absent or delayed latency of SEPs in the pre-hypothermia era were associated with mortality and neurodevelopmental impairment in infants with HIE with PPV 85%–100% (76). However, with the onset of therapeutic hypothermia, other investigators found that infants with bilateral absent SEPs may have a better neurodevelopmental prognosis than previously reported, although the negative predictive value of SEPs for neuroimaging lesions and neurodevelopmental impairment (93%–97%) remains a strength (79, 80). The neurophysiologic tests of VEP and SEP in combination with EEG may further improve prognosis of psychomotor outcome (85% NPV) (77). Measurement of evoked potentials typically occurs after completion of therapeutic hypothermia, which may limit the utility of the technique for early prognostication. These controversial findings have also led clinicians to interpret evoked potentials with caution in the cooled HIE population.

## Multi-modality neuromonitoring with aEEG and NIRS

NIRS and aEEG or cEEG are the neuromonitoring modalities most utilized in NICUs, specifically in newborns with HIE. The use of both modalities has the distinct advantage of simultaneous assessment of both cerebral oxygenation and brain function. NIRS serves as a trend monitor to evaluate the balance between tissue oxygen delivery and consumption while aEEG or EEG provides a continuous monitoring of cerebral function while also alerting the medical providers to electrographic seizure activity. Stratification of hypoxic-ischemic injury may be distinguished by findings on combined neuromonitoring. In several animal models of HIE, NIRS was highly sensitive to an acute hypoxic ischemic insult, while aEEG was less sensitive, particularly for the detection of mild hypoxia ischemia (81, 82). However, NIRS was not a good indicator of severity of evolving injury due to recovery of hemodynamic parameters over time, compared to depressed aEEG amplitudes which persisted after cessation of hypoxia-ischemia and correlated with insult severity (81–84).

In clinical studies of neonates with HIE, combined use of aEEG and NIRS improved predictive value compared to either modality alone for both MRI abnormalities and neurodevelopmental outcomes at 18 months of age or 30 months of age (50, 51, 85). In a cohort of 32 infants cooled for HIE, a combination of reassuring rScO2 and aEEG background pattern at 54–60 h of age led to a combined negative predictive value (NPV) for brain MRI injury of 95% compared to 86% for rScO2 alone or 91% for aEEG pattern alone (85). Positive predictive value for MRI injury was less robust, ranging from 70%–78% after 48 h (85).

**Figure 7:**
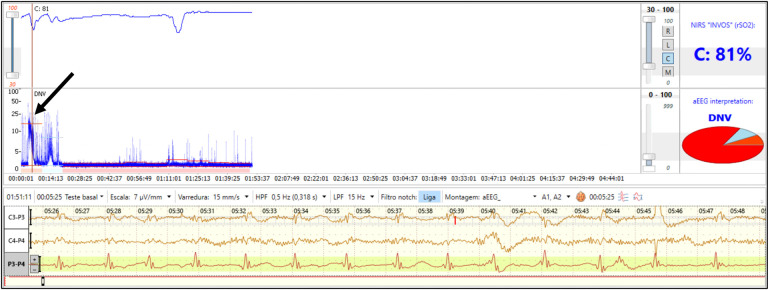
Combined neuromonitoring with aEEG and NIRS in a preterm infant with clinical seizures on the first day of life after severe anemia. The aEEG shows a flat tracing with seizure activity (arrow). Simultaneous NIRS tracing shows supranormal rScO2 Which transiently decreases during seizure activity (reproduced with permission from Variane GFT, Chock VY, Netto A, Pietrobom RFR, Van Meurs KP. Simultaneous Near-Infrared Spectroscopy (NIRS) and Amplitude-Integrated Electroencephalography (aEEG): Dual Use of Brain Monitoring Techniques Improves Our Understanding of Physiology. Front Pediatr. 2019;7:560).

Combining neuromonitoring with NIRS and EEG may also have promise for improved seizure detection and improved assessment of hemodynamic changes during seizure evolution (86). Higher cerebral metabolic demand during a seizure may lead to a decrease in rScO2 (Figure 7) (87). The corresponding degree of cerebral hypoxia may be helpful in classifying seizure severity and evaluating the effectiveness of anticonvulsant therapy.

Simultaneous use of these neuromonitoring techniques may improve the understanding of alterations in cerebral and systemic hemodynamics and the resulting risk of cerebral injury. Some infants with HIE may also require extracorporeal life support (ECLS), and close neuromonitoring in these patients is particularly important given their inherent risk for both hemodynamic instability and abnormal brain function (88, 89). Despite the advantages, the simultaneous use of NIRS and aEEG or cEEG in neonates with HIE has not yet been broadly utilized.

## Comparison of neuromonitoring techniques for prediction of outcome

Selection of optimal neuromonitoring modalities for the infant with HIE must take into consideration sensitivity and specificity for prediction of adverse neurodevelopmental outcomes, timing of monitoring to impact clinical care, and ease of implementation and interpretation at the bedside. Table 2 summarizes this information for the modalities previously reviewed and incorporates some of the available predictive data from several systematic reviews (76, 90, 91). Techniques are frequently compared to later brain MRI performed after completion of therapeutic hypothermia. Imaging with T1/T2 weighted MRI in the first week had high sensitivity (0.89), while diffusion weighted MRI (DWI) had high specificity (0.89) (76). In addition, MRI completed at 4–8 days performed better than later MRI. Injury to the posterior limb of the internal capsule or to the thalami on DWI or increased lactate/N-acetylaspartate peak were strong predictors (91). However, before MRI changes become evident, earlier markers of brain injury are needed for prognostication while undergoing therapeutic hypothermia. Neurologic exam and cerebral ultrasound performed poorly as predictors of outcome, with a large number of false positive findings (76). In contrast, background pattern and presence of seizures on aEEG and EEG are promising predictors of adverse outcomes, with maximal predictive value seen at 48–72 h after birth (Table 2). EEG may have a higher specificity than aEEG, but this needs to be balanced by the complexity and difficulty in performing and interpreting EEG. The modalities of NIRS, HRV, and SEPs need larger studies to confirm predictive ability and thresholds, but in the case of NIRS and HRV, have the potential for earlier discrimination of outcomes. Combining neuromonitoring modalities also may improve prediction and requires further investigation.

**Table 2:** Comparison of neuromonitoring modalities.

Monitoring Technique	Strengths	Limitations	Optimal time period for prediction of outcome	Sensitivity for adverse outcome	Specificity for adverse outcome
cEEG	Continuous, bedside monitor; Gold standard for seizure detection	Labor intensive, specialization needed to apply and interpret EEG	First 72 h after birth	0.92^a^, 0.63^b^, 0.86^c^	0.83^a^, 0.82^b^, 0.58^c^
aEEG	Continuous, bedside monitor; Easy to apply and interpret background patterns. Seizure detection over central and parietal regions	Limited number of channels; May miss short or low amplitude seizures	48–72 h after birth	0.93^a^, 0.90^b^, 0.78^c^	0.90^a^, 0.46^b^, 0.90^c^
NIRS	Continuous, bedside monitor; Measures trends in cerebral oxygenation	Best comparative significance as a trend monitor rather than absolute values	Varying predictive times, but most studies suggest by at least 24 h after birth	0.88^d^, 0.92^e^	0.50^d^, 0.64^e^
HRV	Bedside monitoring of measures of autonomic balance	Typically requires data processing; Mainly a few retrospective studies; Methodologies and definitions inconsistent; Need additional research in HIE	Varying- possibly by 24 h after birth and post-rewarming	0.8^f^, 0.74^g^	0.9^f^, 0.79^g^
VEP	Assess optic pathway	Not a continuous measure; Performed after completion of cooling; Controversial studies of predictive value	After completion of therapeutic hypothermia	0.90^a^, 0.50^h^	0.92^a^, 0.93^h^
SEP	Assess function of sensory cortex and sensory pathways; Good negative predictive value	Not a continuous measure; Performed after completion of cooling; Controversial studies of predictive value	After completion of therapeutic hypothermia	0.93^a^, 0.52^b^	0.78^a^, 0.76^b^

cEEG = continuous electroencephalography, aEEG = amplitude-integrated electroencephalography, NIRS = near-infrared spectroscopy, HRV = heart rate variability, VEP = visual evoked potentials, SEP = somatosensory evoked potentials. TH = therapeutic hypothermia.

^a^ Pooled sensitivity and specificity for death or adverse neurodevelopmental outcome at minimum 18 months, *n* = 1,306 infants with HIE both treated with TH and without TH from 13 studies in systematic review (76).

^b^ Pooled sensitivity and specificity for adverse neurodevelopmental outcome between 18 and 36 months of age, *n* = 1,458 infants with HIE treated with TH from 4 studies in systematic review (90).

^c^ Approximation of pooled sensitivity and specificity for death or adverse neurodevelopmental outcome at minimum 18 months, *n* = 3,072 infants with HIE treated with TH from 37 studies in meta-analysis (91).

^d^ (51).

^e^ (50).

^f^ (68).

^g^ (92).

^h^ (77).

## Conclusions

Neuromonitoring in neonates with HIE plays an important role for both diagnostic and prognostic purposes. As future neuroprotective strategies are developed in the era of therapeutic hypothermia, timely initiation of neuromonitoring is critical for risk stratification of neonates with HIE, early prognostication, and counseling of families. A combination of neuromonitoring techniques with HRV metrics, bedside imaging, evoked potentials, or other newer technologies in the infant with HIE requires further exploration. Multimodality neuromonitoring with aEEG/EEG and NIRS holds particular promise for continuous, bedside assessment of both electrical function and hemodynamic changes in the brain. Consideration should also be given to the necessary training of bedside staff for the implementation of neuromonitoring modalities and interpretation of results. An individualized approach to care using real-time neuromonitoring will ideally optimize neurodevelopmental outcomes in the vulnerable neonatal HIE population.

## References

[1] Jacobs SE Berg M Hunt R Tarnow-Mordi WO Inder TE Davis PG. Cooling for newborns with hypoxic ischaemic encephalopathy. Cochrane Database Syst Rev. (2013) 1:CD003311. 10.1002/14651858.CD003311.pub3 PMC7003568 23440789

[2] Shellhaas RA Chang T Tsuchida T Scher MS Riviello JJ Abend NS The American clinical neurophysiology Society's Guideline on continuous electroencephalography monitoring in neonates. J Clin Neurophysiol. (2011) 28(6):611–7. 10.1097/WNP.0b013e31823e96d7 22146359

[3] Murray DM Boylan GB Ali I Ryan CA Murphy BP Connolly S. Defining the gap between electrographic seizure burden, clinical expression and staff recognition of neonatal seizures. Arch Dis Child Fetal Neonatal Ed. (2008) 93(3):F187–191. 10.1136/adc.2005.086314 17626147

[4] Scher MS Alvin J Gaus L Minnigh B Painter MJ. Uncoupling of EEG-clinical neonatal seizures after antiepileptic drug use. Pediatr Neurol. (2003) 28(4):277–80. 10.1016/S0887-8994(02)00621-5 12849880

[5] Srinivasakumar P Zempel J Trivedi S Wallendorf M Rao R Smith B Treating EEG seizures in hypoxic ischemic encephalopathy: a randomized controlled trial. Pediatrics. (2015) 136(5):e1302–1309. 10.1542/peds.2014-3777 26482675

[6] van Rooij LGM Toet MC van Huffelen AC Groenendaal F Laan W Zecic A Effect of treatment of subclinical neonatal seizures detected with aEEG: randomized, controlled trial. Pediatrics. (2010) 125(2):e358–366. 10.1542/peds.2009-0136 20100767

[7] Bashir RA Espinoza L Vayalthrikkovil S Buchhalter J Irvine L Bello-Espinosa L Implementation of a neurocritical care program: improved seizure detection and decreased antiseizure medication at discharge in neonates with hypoxic-ischemic encephalopathy. Pediatr Neurol. (2016) 64:38–43. 10.1016/j.pediatrneurol.2016.07.007 27647155

[8] Harris ML Malloy KM Lawson SN Rose RS Buss WF Mietzsch U. Standardized treatment of neonatal Status epilepticus improves outcome. J Child Neurol. (2016) 31(14):1546–54. 10.1177/0883073816664670 27581850

[9] Wietstock SO Bonifacio SL McCulloch CE Kuzniewicz MW Glass HC. Neonatal neurocritical care service Is associated with decreased administration of seizure medication. J Child Neurol. (2015) 30(9):1135–41. 10.1177/0883073814553799 25380602 PMC4424192

[10] Wietstock SO Bonifacio SL Sullivan JE Nash KB Glass HC. Continuous video electroencephalographic (EEG) monitoring for electrographic seizure diagnosis in neonates: a single-center study. J Child Neurol. (2016) 31(3):328–32. 10.1177/0883073815592224 26129976 PMC4696927

[11] Glass HC Glidden D Jeremy RJ Barkovich AJ Ferriero DM Miller SP. Clinical neonatal seizures Are independently associated with outcome in infants at risk for hypoxic-ischemic brain injury. J Pediatr. (2009) 155(3):318–23. 10.1016/j.jpeds.2009.03.040 19540512 PMC3014109

[12] Wusthoff CJ Dlugos DJ Gutierrez-Colina A Wang A Cook N Donnelly M Electrographic seizures during therapeutic hypothermia for neonatal hypoxic-ischemic encephalopathy. J Child Neurol. (2011) 26(6):724–8. 10.1177/0883073810390036 21447810 PMC3102150

[13] Glass HC Wusthoff CJ Comstock BA Numis AL Gonzalez FF Maitre N Risk of seizures in neonates with hypoxic-ischemic encephalopathy receiving hypothermia plus erythropoietin or placebo. Pediatr Res. (2022). 10.1038/s41390-022-02398-w. [online ahead of print] PMC10239788 36470964

[14] Chalak LF Pappas A Tan S Das A Sánchez PJ Laptook AR Association between increased seizures during rewarming after hypothermia for neonatal hypoxic ischemic encephalopathy and abnormal neurodevelopmental outcomes at 2-year follow-up: a nested multisite cohort study. JAMA Neurol. (2021) 78(12):1484–93. 10.1001/jamaneurol.2021.3723 34882200 PMC8524352

[15] Tsuchida TN. EEG Background patterns and prognostication of neonatal encephalopathy in the era of hypothermia. J Clin Neurophysiol. (2013) 30(2):122–5. 10.1097/WNP.0b013e3182872ac2 23545762

[16] Glass HC Shellhaas RA Wusthoff CJ Chang T Abend NS Chu CJ Contemporary profile of seizures in neonates: a prospective cohort study. J Pediatr. (2016) 174:98–103.e1. 10.1016/j.jpeds.2016.03.035 27106855 PMC4925241

[17] Koskela T Kendall GS Memon S Sokolska M Mabuza T Huertas-Ceballos A Prognostic value of neonatal EEG following therapeutic hypothermia in survivors of hypoxic-ischemic encephalopathy. Clin Neurophysiol. (2021) 132(9):2091–100. 10.1016/j.clinph.2021.05.031 34284244 PMC8407358

[18] Low E Boylan GB Mathieson SR Murray DM Korotchikova I Stevenson NJ Cooling and seizure burden in term neonates: an observational study. Arch Dis Child Fetal Neonatal Ed. (2012) 97(4):F267–272. 10.1136/archdischild-2011-300716 22215799

[19] Hellstrom-Westas L. Continuous electroencephalography monitoring of the preterm infant. Clin Perinatol. (2006) 33(3):633–47., vi. 10.1016/j.clp.2006.06.003 16950316

[20] al Naqeeb N Edwards AD Cowan FM Azzopardi D. Assessment of neonatal encephalopathy by amplitude-integrated electroencephalography. Pediatrics. (1999) 103(6 Pt 1):1263–71. 10.1542/peds.103.6.1263 10353940

[21] Hellström-Westas L Rosén I de Vries L Greisen G. Amplitude-integrated EEG classification and interpretation in preterm and term infants. Neoreviews. (2006) 7(2):376–e86. 10.1542/neo.7-2-e76

[22] Bjerre I Hellström-Westas L Rosén I Svenningsen N. Monitoring of cerebral function after severe asphyxia in infancy. Arch Dis Child. (1983) 58(12):997–1002. 10.1136/adc.58.12.997 6660900 PMC1628591

[23] ter Horst HJ Sommer C Bergman KA Fock JM van Weerden TW Bos AF. Prognostic significance of amplitude-integrated EEG during the first 72 h after birth in severely asphyxiated neonates. Pediatr Res. (2004) 55(6):1026–33. 10.1203/01.pdr.0000127019.52562.8c 15155870

[24] Gluckman PD Wyatt JS Azzopardi D Ballard R Edwards AD Ferriero DM Selective head cooling with mild systemic hypothermia after neonatal encephalopathy: multicentre randomised trial. Lancet. (2005) 365(9460):663–70. 10.1016/S0140-6736(05)17946-X 15721471

[25] Azzopardi DV Strohm B Edwards AD Dyet L Halliday HL Juszczak E Moderate hypothermia to treat perinatal asphyxial encephalopathy. N Engl J Med. (2009) 361(14):1349–58. 10.1056/NEJMoa0900854 19797281

[26] Simbruner G Mittal RA Rohlmann F Muche R. Neo.nEURO.network trial participants. Systemic hypothermia after neonatal encephalopathy: outcomes of neo.nEURO.network RCT. Pediatrics. (2010) 126(4):e771–778. 10.1542/peds.2009-2441 20855387

[27] Thoresen M Hellström-Westas L Liu X de Vries LS. Effect of hypothermia on amplitude-integrated electroencephalogram in infants with asphyxia. Pediatrics. (2010) 126(1):e131–139. 10.1542/peds.2009-2938 20566612

[28] Sewell EK Vezina G Chang T Tsuchida T Harris K Ridore M Evolution of amplitude-integrated electroencephalogram as a predictor of outcome in term encephalopathic neonates receiving therapeutic hypothermia. Am J Perinatol. (2018) 35(3):277–85. 10.1055/s-0037-1607212 28958093 PMC7863699

[29] Spitzmiller RE Phillips T Meinzen-Derr J Hoath SB. Amplitude-integrated EEG Is useful in predicting neurodevelopmental outcome in full-term infants with hypoxic-ischemic encephalopathy: a meta-analysis. J Child Neurol. (2007) 22(9):1069–78. 10.1177/0883073807306258 17890403

[30] Awal MA Lai MM Azemi G Boashash B Colditz PB. EEG Background features That predict outcome in term neonates with hypoxic ischaemic encephalopathy: a structured review. Clin Neurophysiol. (2016) 127(1):285–96. 10.1016/j.clinph.2015.05.018 26105684

[31] Del Río R Ochoa C Alarcon A Arnáez J Blanco D García-Alix A. Amplitude integrated electroencephalogram as a prognostic tool in neonates with hypoxic-ischemic encephalopathy: a systematic review. PLoS ONE. (2016) 11(11):e0165744. 10.1371/journal.pone.0165744 27802300 PMC5089691

[32] Chandrasekaran M Chaban B Montaldo P Thayyil S. Predictive value of amplitude-integrated EEG (aEEG) after rescue hypothermic neuroprotection for hypoxic ischemic encephalopathy: a meta-analysis. J Perinatol. (2017) 37(6):684–9. 10.1038/jp.2017.14 28252661

[33] Hellstrom-Westas L Rosen I Svenningsen NW. Predictive value of early continuous amplitude integrated EEG recordings on outcome after severe birth asphyxia in full term infants. Arch Dis Child Fetal Neonatal Ed. (1995) 72(1):F34–8. 10.1136/fn.72.1.F34 7743282 PMC2528413

[34] Dix LML van Bel F Baerts W Lemmers PMA. Comparing near-infrared spectroscopy devices and Their sensors for monitoring regional cerebral oxygen saturation in the neonate. Pediatr Res. (2013) 74(5):557–63. 10.1038/pr.2013.133 23942560

[35] Alderliesten T Dix L Baerts W Caicedo A van Huffel S Naulaers G Reference values of regional cerebral oxygen saturation during the first 3 days of life in preterm neonates. Pediatr Res. (2016) 79(1–1):55–64. 10.1038/pr.2015.186 26389823

[36] Chock VY Smith E Tan S Ball MB Das A Hintz SR Early brain and abdominal oxygenation in extremely low birth weight infants. Pediatr Res. (2022) 92(4):1034–41. 10.1038/s41390-022-02082-z 35513716 PMC9588487

[37] McNeill S Gatenby JC McElroy S Engelhardt B. Normal cerebral, renal and abdominal regional oxygen saturations using near-infrared spectroscopy in preterm infants. J Perinatol. (2011) 31(1):51–7. 10.1038/jp.2010.71 20539273 PMC3013378

[38] Bailey SM Hendricks-Munoz KD Mally P. Cerebral, renal, and splanchnic tissue oxygen saturation values in healthy term newborns. Am J Perinatol. (2014) 31(4):339–44. 10.1055/s-0033-1349894 23873114

[39] Bernal NP Hoffman GM Ghanayem NS Arca MJ. Cerebral and somatic near-infrared spectroscopy in normal newborns. J Pediatr Surg. (2010) 45(6):1306–10. 10.1016/j.jpedsurg.2010.02.110 20620336

[40] Garvey AA Pavel AM Murray DM Boylan GB Dempsey EM. Does Early cerebral near-infrared spectroscopy monitoring predict outcome in neonates with hypoxic ischaemic encephalopathy? A systematic review of diagnostic test accuracy. Neonatology. (2021)119(1):1–9. 10.1159/000518687 34818237

[41] Yenari MA Han HS. Neuroprotective mechanisms of hypothermia in brain ischaemia. Nat Rev Neurosci. (2012) 13(4):267–78. 10.1038/nrn3174 22353781

[42] Garvey AA O’Toole JM Livingstone V Walsh B Moore M Pavel AM Evolution of early cerebral NIRS in hypoxic ischaemic encephalopathy. Acta Paediatr. (2022) 111(10):1870–7. 10.1111/apa.16493 35869794 PMC9545024

[43] Chock VY Frymoyer A Yeh CG Van Meurs KP. Renal saturation and acute kidney injury in neonates with hypoxic ischemic encephalopathy undergoing therapeutic hypothermia. J Pediatr. (2018) 200:232–239.e1. 10.1016/j.jpeds.2018.04.076 29866591

[44] Jain SV Pagano L Gillam-Krakauer M Slaughter JC Pruthi S Engelhardt B. Cerebral regional oxygen saturation trends in infants with hypoxic-ischemic encephalopathy. Early Hum Dev. (2017) 113:55–61. 10.1016/j.earlhumdev.2017.07.008 28772198

[45] Peng S Boudes E Tan X Saint-Martin C Shevell M Wintermark P. Does near-infrared spectroscopy identify asphyxiated newborns at risk of developing brain injury during hypothermia treatment? Am J Perinatol. (2015) 32(6):555–64. 10.1055/s-0034-1396692 25594221

[46] Massaro AN Bouyssi-Kobar M Chang T Vezina LG du Plessis AJ Limperopoulos C. Brain perfusion in encephalopathic newborns after therapeutic hypothermia. AJNR Am J Neuroradiol. (2013) 34(8):1649–55. 10.3174/ajnr.A3422 23493898 PMC4376377

[47] Szakmar E Smith J Yang E Volpe JJ Inder T El-Dib M. Association between cerebral oxygen saturation and brain injury in neonates receiving therapeutic hypothermia for neonatal encephalopathy. J Perinatol. (2021) 41(2):269–77. 10.1038/s41372-020-00910-w 33462339

[48] Shellhaas RA Thelen BJ Bapuraj JR Burns JW Swenson AW Christensen MK Limited short-term prognostic utility of cerebral NIRS during neonatal therapeutic hypothermia. Neurology. (2013) 81(3):249–55. 10.1212/WNL.0b013e31829bfe41 23771483 PMC3770165

[49] Ancora G Maranella E Grandi S Sbravati F Coccolini E Savini S Early predictors of short term neurodevelopmental outcome in asphyxiated cooled infants. A combined brain amplitude integrated electroencephalography and near infrared spectroscopy study. Brain Dev. (2013) 35(1):26–31. 10.1016/j.braindev.2011.09.008 22082686

[50] Lemmers PM Zwanenburg RJ Benders MJ de Vries LS Groenendaal F van Bel F Cerebral oxygenation and brain activity after perinatal asphyxia: does hypothermia change Their prognostic value? Pediatr Res. (2013) 74(2):180–5. 10.1038/pr.2013.84 23728382

[51] Niezen CK Bos AF Sival DA Meiners LC Ter Horst HJ. Amplitude-Integrated EEG and cerebral near-infrared spectroscopy in cooled, asphyxiated infants. Am J Perinatol. (2018) 35(9):904–10. 10.1055/s-0038-1626712 29421831

[52] Wintermark P Hansen A Warfield SK Dukhovny D Soul JS. Near-infrared spectroscopy versus magnetic resonance imaging to study brain perfusion in newborns with hypoxic-ischemic encephalopathy treated with hypothermia. Neuroimage. (2014) 85(Pt 1):287–93. 10.1016/j.neuroimage.2013.04.072 23631990 PMC4487623

[53] Massaro AN Govindan RB Vezina G Chang T Andescavage NN Wang Y Impaired cerebral autoregulation and brain injury in newborns with hypoxic-ischemic encephalopathy treated with hypothermia. J Neurophysiol. (2015) 114(2):818–24. 10.1152/jn.00353.2015 26063779 PMC4533061

[54] Howlett JA Northington FJ Gilmore MM Tekes A Huisman TAGM Parkinson C Cerebrovascular autoregulation and neurologic injury in neonatal hypoxic-ischemic encephalopathy. Pediatr Res. (2013) 74(5):525–35. 10.1038/pr.2013.132 23942555 PMC3954983

[55] Chalak LF Zhang R. New wavelet neurovascular bundle for bedside evaluation of cerebral autoregulation and neurovascular coupling in newborns with hypoxic-ischemic encephalopathy. Dev Neurosci. (2017) 39(1–4):89–96. 10.1159/000457833 28355608 PMC5519424

[56] Tian F Sepulveda P Kota S Liu Y Das Y Liu H Regional heterogeneity of cerebral hemodynamics in mild neonatal encephalopathy measured with multichannel near-infrared spectroscopy. Pediatr Res. (2021) 89(4):882–8. 10.1038/s41390-020-0992-5 32492696

[57] Dehaes M Aggarwal A Lin PY Rosa Fortuno C Fenoglio A Roche-Labarbe N Cerebral oxygen metabolism in neonatal hypoxic ischemic encephalopathy during and after therapeutic hypothermia. J Cereb Blood Flow Metab. (2014) 34(1):87–94. 10.1038/jcbfm.2013.165 24064492 PMC3887346

[58] Bersani I Piersigilli F Gazzolo D Campi F Savarese I Dotta A Heart rate variability as possible marker of brain damage in neonates with hypoxic ischemic encephalopathy: a systematic review. Eur J Pediatr. (2021) 180(5):1335–45. 10.1007/s00431-020-03882-3 33245400 PMC7691422

[59] Twomey E Twomey A Ryan S Murphy J Donoghue VB. MR Imaging of term infants with hypoxic-ischaemic encephalopathy as a predictor of neurodevelopmental outcome and late MRI appearances. Pediatr Radiol. (2010) 40(9):1526–35. 10.1007/s00247-010-1692-9 20512322

[60] Massaro AN Campbell HE Metzler M Al-Shargabi T Wang Y du Plessis A Effect of temperature on heart rate variability in neonatal ICU patients with hypoxic-ischemic encephalopathy. Pediatr Crit Care Med. (2017) 18(4):349–54. 10.1097/PCC.0000000000001094 28198757 PMC5402340

[61] Vesoulis ZA Rao R Trivedi SB Mathur AM. The effect of therapeutic hypothermia on heart rate variability. J Perinatol. (2017) 37(6):679–83. 10.1038/jp.2017.42 28383534 PMC5446282

[62] Andersen M Andelius TCK Pedersen MV Kyng KJ Henriksen TB. Severity of hypoxic ischemic encephalopathy and heart rate variability in neonates: a systematic review. BMC Pediatr. (2019) 19(1):242. 10.1186/s12887-019-1603-7 31324176 PMC6639904

[63] Kozár M Javorka K Javorka M Matasová K Zibolen M. Changes of cardiovascular regulation during rewarming in newborns undergoing whole-body hypothermia. Neuro Endocrinol Lett. (2015) 36(5):434–8. 26707043

[64] Aliefendioğlu D Doğru T Albayrak M Dibekmısırlıoğlu E Sanlı C. Heart rate variability in neonates with hypoxic ischemic encephalopathy. Indian J Pediatr. (2012) 79(11):1468–72. 10.1007/s12098-012-0703-2 22359196

[65] Goulding RM Stevenson NJ Murray DM Livingstone V Filan PM Boylan GB. Heart rate variability in hypoxic ischemic encephalopathy during therapeutic hypothermia. Pediatr Res. (2017) 81(4):609–15. 10.1038/pr.2016.245 27855152

[66] Vergales BD Zanelli SA Matsumoto JA Goodkin HP Lake DE Moorman JR Depressed heart rate variability Is associated with abnormal EEG, MRI, and death in neonates with hypoxic ischemic encephalopathy. Am J Perinatol. (2014) 31(10):855–62. 10.1055/s-0033-1361937 24347263 PMC10995890

[67] Metzler M Govindan R Al-Shargabi T Vezina G Andescavage N Wang Y Pattern of brain injury and depressed heart rate variability in newborns with hypoxic ischemic encephalopathy. Pediatr Res. (2017) 82(3):438–43. 10.1038/pr.2017.94 28376079 PMC5570625

[68] Massaro AN Govindan RB Al-Shargabi T Andescavage NN Metzler M Chang T Heart rate variability in encephalopathic newborns during and after therapeutic hypothermia. J Perinatol. (2014) 34(11):836–41. 10.1038/jp.2014.108 24921413 PMC4216618

[69] Suppiej A Vedovelli L Boschiero D Bolzon M Cainelli E. Abnormal heart rate variability at school age in survivors of neonatal hypoxic-ischemic encephalopathy managed with therapeutic hypothermia. Eur J Paediatr Neurol. (2020) 29:66–70. 10.1016/j.ejpn.2020.08.004 32863129

[70] Kayton A DeGrazia M Sharpe E Smith D Perez JA Weiss MD. Correlation between heart rate characteristic Index score and severity of brain injury in neonates with hypoxic-ischemic encephalopathy. Adv Neonatal Care. (2020) 20(4):E70–82. 10.1097/ANC.0000000000000686 31895138

[71] Griffin MP O'Shea TM Bissonette EA Harrell FE Lake DE Moorman JR. Abnormal heart rate characteristics preceding neonatal sepsis and sepsis-Like illness. Pediatr Res. (2003) 53(6):920–6. 10.1203/01.PDR.0000064904.05313.D2 12646726

[72] Yasova Barbeau D Krueger C Huene M Copenhaver N Bennett J Weaver M Heart rate variability and inflammatory markers in neonates with hypoxic-ischemic encephalopathy. Physiol Rep. (2019) 7(15):e14110. 10.14814/phy2.14110 31397094 PMC6687857

[73] Merchant N Azzopardi D. Early predictors of outcome in infants treated with hypothermia for hypoxic-ischaemic encephalopathy. Dev Med Child Neurol. (2015) 57(Suppl 3):8–16. 10.1111/dmcn.12726 25800487

[74] Sanislow W Singh E Yang E Inder T El-Dib M. Value of cranial ultrasound at initiation of therapeutic hypothermia for neonatal encephalopathy. J Perinatol. (2022) 42(3):335–40. 10.1038/s41372-021-01233-0 34663900

[75] Schindler T Stevenson G Jayatilake S Gilbert Y Oei JL Welsh A. Reference ranges for neonatal basal ganglia perfusion as measured by fractional moving blood volume. Neonatology. (2016) 109(2):91–6. 10.1159/000441466 26583917

[76] van Laerhoven H de Haan TR Offringa M Post B van der Lee JH. Prognostic tests in term neonates with hypoxic-ischemic encephalopathy: a systematic review. Pediatrics. (2013) 131(1):88–98. 10.1542/peds.2012-1297 23248219

[77] Cainelli E Trevisanuto D Cavallin F Manara R Suppiej A. Evoked potentials predict psychomotor development in neonates with normal MRI after hypothermia for hypoxic-ischemic encephalopathy. Clin Neurophysiol. (2018) 129(6):1300–6. 10.1016/j.clinph.2018.03.043 29689487

[78] Kamino D Almazrooei A Pang EW Widjaja E Moore AM Chau V Abnormalities in evoked potentials associated with abnormal glycemia and brain injury in neonatal hypoxic-ischemic encephalopathy. Clin Neurophysiol. (2021) 132(1):307–13. 10.1016/j.clinph.2020.09.024 33158762 PMC7855101

[79] Garfinkle J Sant’Anna GM Rosenblatt B Majnemer A Wintermark P Shevell MI. Somatosensory evoked potentials in neonates with hypoxic-ischemic encephalopathy treated with hypothermia. Eur J Paediatr Neurol. (2015) 19(4):423–8. 10.1016/j.ejpn.2015.03.001 25814390

[80] Arriaga-Redondo M Bravo DB Del Hoyo AA Arrondo AP Martín YR Sánchez-Luna M. Prognostic value of somatosensory-evoked potentials in the newborn with hypoxic-ischemic encephalopathy after the introduction of therapeutic hypothermia. Eur J Pediatr. (2022) 181(4):1609–18. 10.1007/s00431-021-04336-0 35066625

[81] Zhang D Hou X Liu Y Zhou C Luo Y Ding H. The utility of amplitude-integrated EEG and NIRS measurements as indices of hypoxic ischaemia in the newborn pig. Clin Neurophysiol. (2012) 123(8):1668–75. 10.1016/j.clinph.2011.10.051 22277760

[82] Tichauer KM Elliott JT Hadway JA Lee TY St Lawrence K. Cerebral metabolic rate of oxygen and amplitude-integrated electroencephalography during early reperfusion after hypoxia-ischemia in piglets. J Appl Physiol. (2009) 106(5):1506–12. 10.1152/japplphysiol.91156.2008 19299571

[83] Gavilanes AW Vles JS von Siebenthal K Reulen JP Nieman FH van Sprundel R Electrocortical brain activity, cerebral haemodynamics and oxygenation during progressive hypotension in newborn piglets. Clin Neurophysiol. (2001) 112(1):52–9. 10.1016/S1388-2457(00)00499-5 11137661

[84] Thoresen M Haaland K Løberg EM Whitelaw A Apricena F Hankø E A piglet survival model of posthypoxic encephalopathy. Pediatr Res. (1996) 40(5):738–48. 10.1203/00006450-199611000-00014 8910940

[85] Goeral K Urlesberger B Giordano V Kasprian G Wagner M Schmidt L Prediction of outcome in neonates with hypoxic-ischemic encephalopathy II: role of amplitude-integrated electroencephalography and cerebral oxygen saturation measured by near-infrared spectroscopy. Neonatology. (2017) 112(3):193–202. 10.1159/000468976 28704822

[86] Wallois F Patil A Héberlé C Grebe R. EEG-NIRS in epilepsy in children and neonates. Neurophysiol Clin. (2010) 40(5–6):281–92. 10.1016/j.neucli.2010.08.004 21093799

[87] Variane GFT Chock VY Netto A Pietrobom RFR Van Meurs KP. Simultaneous near-infrared spectroscopy (NIRS) and amplitude-integrated electroencephalography (aEEG): dual use of brain monitoring techniques improves Our understanding of physiology. Front Pediatr. (2019) 7:560. 10.3389/fped.2019.00560 32039117 PMC6985148

[88] Clair MP Rambaud J Flahault A Guedj R Guilbert J Guellec I Prognostic value of cerebral tissue oxygen saturation during neonatal extracorporeal membrane oxygenation. PLoS One. (2017) 12(3):e0172991. 10.1371/journal.pone.0172991 28278259 PMC5344369

[89] Lin JJ Banwell BL Berg RA Dlugos DJ Ichord RN Kilbaugh TJ Electrographic seizures in children and neonates undergoing extracorporeal membrane oxygenation. Pediatr Crit Care Med. (2017) 18(3):249–57. 10.1097/PCC.0000000000001067 28099234 PMC5336402

[90] Liu W Yang Q Wei H Dong W Fan Y Hua Z. Prognostic value of clinical tests in neonates with hypoxic-ischemic encephalopathy treated with therapeutic hypothermia: a systematic review and meta-analysis. Front Neurol. (2020) 11:133. 10.3389/fneur.2020.00133 32161566 PMC7052385

[91] Ouwehand S Smidt LCA Dudink J Benders MJNL de Vries LS Groenendaal F Predictors of outcomes in hypoxic-ischemic encephalopathy following hypothermia: a meta-analysis. Neonatology. (2020) 117(4):411–27. 10.1159/000505519 32235122

[92] Oliveira V Martins R Liow N Teiserskas J von Rosenberg W Adjei T Prognostic accuracy of heart rate variability analysis in neonatal encephalopathy: a systematic review. Neonatology. (2019) 115(1):59–67. 10.1159/000493002 30300885

